# Impact of maternal dietary fat supplementation during gestation upon skeletal muscle in neonatal pigs

**DOI:** 10.1186/s12899-014-0006-0

**Published:** 2014-08-27

**Authors:** Hernan P Fainberg, Kayleigh L Almond, Dongfang Li, Cyril Rauch, Paul Bikker, Michael E Symonds, Alison Mostyn

**Affiliations:** School of Veterinary Medicine and Science, University of Nottingham, Sutton Bonington Campus, Leicestershire, LE12 5RD UK; Early Life Nutrition Research Unit, Academic Child Health, School of Clinical Sciences, University Hospital, The University of Nottingham, Nottingham, NG7 2UH UK; School of Biosciences, The University of Nottingham, Sutton Bonington Campus, Leicestershire, LE12 5RD UK; Schothorst Feed Research, PO Box 533, 8200 AM Lelystad, The Netherlands; Current address: Primary Diets, Melmerby Industrial state, Melmerby, Ripon, North Yorkshire, HG4 5HP UK; Current address: Wageningen UR Livestock Research, PO Box 338, 6700 AH Wageningen, The Netherlands

**Keywords:** Nutrition, Muscle, Fetal development, Growth

## Abstract

**Background:**

Maternal diet during pregnancy can modulate skeletal muscle development of the offspring. Previous studies in pigs have indicated that a fat supplemented diet during pregnancy can improve piglet outcome, however, this is in contrast to human studies suggesting adverse effects of saturated fats during pregnancy. This study aimed to investigate the impact of a fat supplemented (palm oil) “high fat” diet on skeletal muscle development in a porcine model. Histological and metabolic features of the *biceps femoris* muscle obtained from 7-day-old piglets born to sows assigned to either a commercial (C, n = 7) or to an isocaloric fat supplementation diet (“high fat” HF, n = 7) during pregnancy were assessed.

**Results:**

Offspring exposed to a maternal HF diet demonstrated enhanced muscular development, reflected by an increase in fractional growth rate, rise in myofibre cross-sectional area, increased storage of glycogen and reduction in lipid staining of myofibres. Although both groups had similar intramuscular protein and triglyceride concentrations, the offspring born to HF mothers had a higher proportion of arachidonic acid (C20:4n6) and a reduction in α-linolenic acid (C18:3n3) compared to C group offspring. The HF group muscle also exhibited a higher ratio of C20:3n6 to C20:4n6 and total n-6 to n-3 in conjunction with up-regulation of genes associated with free fatty acid uptake and biogenesis.

**Conclusion:**

In conclusion, a HF gestational diet accelerates the maturation of offspring *biceps femoris* muscle, reflected in increased glycolytic metabolism and fibre cross sectional area, differences accompanied with a potential resetting of myofibre nutrient uptake.

## Background

Despite the success of breeding programs for increasing lean mass, the rate of postnatal death in European “commercial” pig breeds (such as the Large White) has remained high at approximately 10-20% per litter and is therefore a major economic concern to the swine industry [1-3]. The neonatal piglet has body energy reserves of around 270 KJ, mainly in the form of glycogen located in the liver and muscle with little white adipose tissue (~1% body weight comprises adipose tissue) and currently little evidence for brown adipose tissue [4]. Consequently the neonatal pig is reliant on a regular supply of milk to prevent hypoglycaemia, a major cause of neonatal death [3,5,6]. Consequently each dam requires a daily intake of up to 1340 KJ per day to maintain a positive energy balance and support the nutritional requirements of her litter [5,7].

One strategy to reduce neonatal mortality is to add saturated fat to the maternal diet during gestation to promote glycogen and fat deposition in the fetus [8-12] and to promote pre-weaning survival of piglets by improving their energy status [12]. However, there is evidence in both human and animal studies to suggest that consumption of a high-fat diet increases the risk of developing insulin resistance, sometimes without an increase in fat mass [13-19].

The ability to suckle effectively during the first week of postnatal life is critical to piglet survival, highlighting the importance of physical mobility and therefore appropriate muscular development [3,20]. Formation and contractile differentiation of muscle fibres in the pig, as in most large mammals including humans and sheep, occurs in distinct developmental stages during gestation [21,22] and can be influenced by external factors, including the maternal and postnatal nutritional environments [23,24]. Primary myofibres begin to differentiate at around 35 days gestation, followed by secondary and tertiary fibres and by the first days of postnatal life this process is permanently inhibited [22]. Between 75 days gestation until 8 weeks of postnatal life, each fibre develops its own metabolic phenotype, which is associated with muscular maturation [21,25]. However, during the first 7 days of postnatal life, due to improvements in fatty acid metabolism, there is a rapid maturation in the piglets’ skeletal muscle fibre types and growth which requires high levels of protein synthesis [22,26]. Between 7 and 26 days (weaning) protein synthesis declines rapidly [27]. This maturation is characterised by a rise in glycogen storage, leading to an increase in glycolytic metabolism, gene expression plus the activation of enzymes involved in carbohydrate metabolism [28]. These metabolic changes induce fibre hypertrophy and increase muscular strength – both changes are necessary to increase body weight and facilitate mobility and food intake [25,27,28]. Modifications in myofibre development during the first weeks of postnatal life could therefore have important implications for subsequent muscular metabolic capacity. The n-3 (α linolenic acid) and n-6 (linoleic acid) polyunsaturated fatty acids (PUFAs) are considered to be “essential” as they cannot be synthesised by mammalian cells [29], these fatty acids are a crucial component of the phospholipid membrane of skeletal muscle fibres. Whole animal and in vitro studies have highlighted the importance of essential fatty acids in muscle development; improved muscle development, maintenance and function was observed in cattle fed an n-3 PUFA supplemented diet and n-3 PUFA supplementation of L6 skeletal muscle cells also activated differentiation [30,31]. Previous work conducted by our research team, in which we compared the hepatic development of new-born Large White pigs with the slower growing Meishan breed, revealed that these essential fatty acids have a major role in hepatic development during gestation and postnatal growth and metabolism [32]. The ratios of n-6 and n-3 fatty acids within the membrane of skeletal muscle have been implicated in glucose homeostasis [8]. To examine the underlying metabolic and developmental changes during early life, the present study compared two maternal isocaloric gestational diets, a standard commercial diet (control; C) or palmitic acid (a saturated fatty acid) supplemented diet (high fat; HF) upon the *biceps femoris*, a mainly glycolytic muscle, of 7-day-old offspring, when energy requirements are at a peak. Given the impact of fatty acids on muscle development outlined above, we hypothesise that increases in maternal fat intake during gestation would accelerate the *biceps femoris’* maturation from oxidative to glycolytic metabolism; increase energy usage and related parameters of muscular development, including myofibre hypertrophy as well as produce differential fatty acid profiles.

## Results

### Characteristics of the sows and their offspring

**Figure 1:**
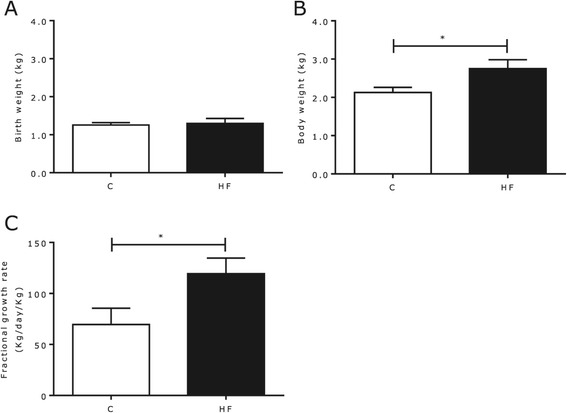
**Influence of maternal nutrition throughout gestation on early postnatal development. (A)** birth weight, **(B)** weight on day 7 of postnatal age and **(C)** fractional growth rate as observed in control (white; n = 7) and high fat (black; n = 7) piglets. Bar graphs illustrate means ± SEM (* p < 0.05).

Sow body weight increased throughout gestation, irrespective of diet (P < 0.05, Table 1). Maternal plasma HDL concentrations were raised in the HF group compared to controls, whereas plasma LDL, triglycerides and glucose were unaffected. Sow’s milk on day 2 of lactation contained 8% fat, 5% lactose and 6% protein and was unaffected by maternal diet [33]. There was no effect on the length of gestation (C = 117.4 ± 0.3; HF = 116.1 ± 0.7 days (P = 0.19)), mean birth weight (C = 1.22 ± 0.04; HF = 1.25 ± 0.07 kg (P = 0.56)) or litter size (C, 14.9 ± 0.8; FS, 16.4 ± 0.7 piglets/sow (P = 0.07)), but by 7 days of age the offspring born to HF mothers were heavier than controls evidenced by a greater fractional growth rate (Figure 1).

**Table 1:** **Body weight and blood biochemistry of the sows as measured at 0, 40 and 108 days of gestation**

**Days of gestation**	**0**	**40**	**108**	**p**
Sow weight (Kg)				
C	215.5 ± 13.4	236.6 ± 11.8	287.7 ± 10.5	
HF	209.8 ± 9.2	254.6 ± 8.6	281.8 ± 8.7	
HDL (mmol/l)				
C	0.55 ± 0.05	0.55 ± 0.05*	0.42 ± 0.03^#^	_*_ < 0.05; # < 0.01
HF	0.66 ± 0.05	0.77 ± 0.04*	0.56 ± 0.04^#^	
LDL (mmol/l)				
C	0.68 ± 0.06	0.87 ± 0.05	0.74 ± 0.03	
HF	0.75 ± 0.06	0.98 ± 0.08	0.84 **±** 0.08	
Glucose (mmol/l)				
C	3.88 ± 0.57	3.93 ± 0.12	3.93 ± 0.10	
HF	3.58 ± 0.51	4.11 ± 0.08	3.70 ± 0.17	

Values are means ± SEM. C: control (n = 7); HF: high fat (n = 7); HDL: High-density lipoprotein; LDL: Low-density lipoprotein. Superscripts denote significance levels between dietary groups, _*_ < 0.05; # < 0.01.

### Fibre characteristics and association with body weight

**Figure 2:**
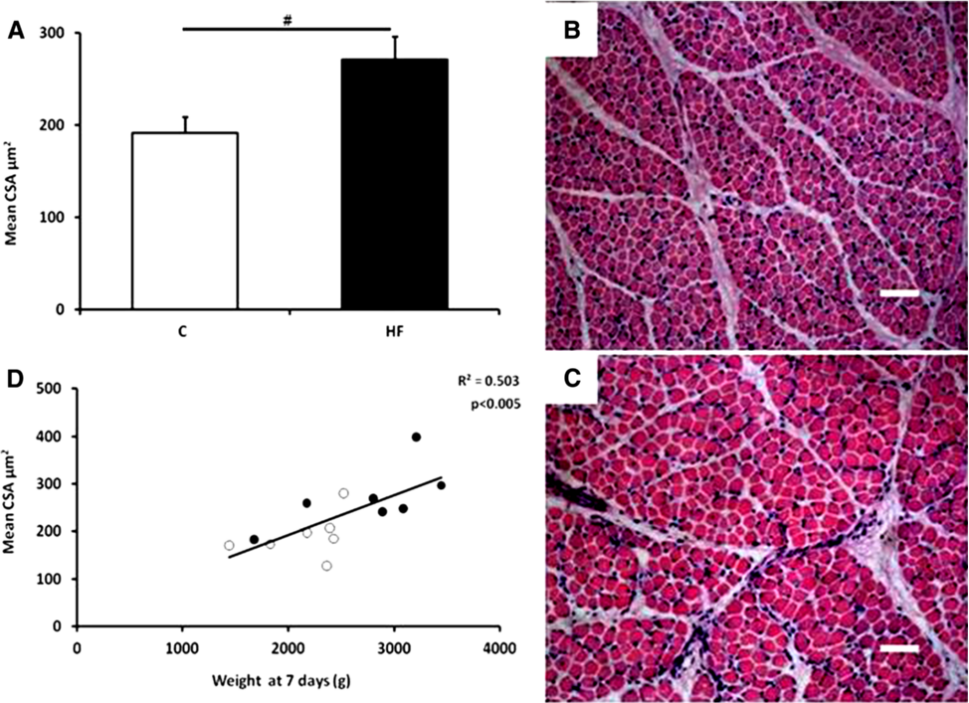
**The influence of maternal diet during gestation on myofibre development in 7-day old offspring. (A)** Quantitative analysis of myofibre cross sectional areas (CSA) in the *biceps femoris* in control (white; n = 7) and high fat (black; n = 7) piglets. Bar graphs illustrate means ± SEM (# p < 0.05). Histological images of the *biceps femoris* fibres generated by hematoxylin and eosin (**B**: control group; **C**: HF group, scale bar = 50 μm). **(D)** The relationship between mean CSA and body weight at 7 days of age in control (white; n = 7) and high fat (black; n = 7) offspring.

**Figure 3:**
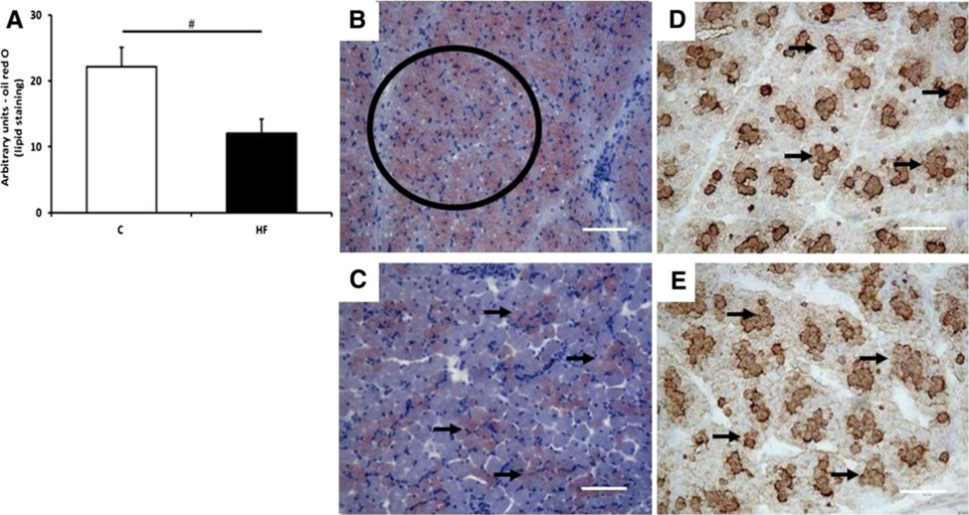
**The effect of maternal fat supplementation on offspring intramyofibre lipid deposition. (A)** Bar graph illustrating quantitative analysis of oil red O staining of intramyofibre lipid deposition in 7-day-old offspring exposed control (white; n = 7) and high fat (black; n = 7) piglets (means ± SEM (# p < 0.05)). Histological images of the *biceps femoris* fibres generated by oil red O staining (**B**: control; **C**: HF group) and anti-slow MyHC staining (**D**: control; **E**: HF group). Circle outlines area of interest for analysis, arrows indicate the lipid stain in relation to type I fibres. Scale bar = 50 μm.

Myofibre cross sectional area (CSA) was higher in the HF group (Figure 2A, B and C), this was positively associated with body weight at 7 days of age as demonstrated in Figure 2D. HF offspring also had reduced myocellular lipids; these were mainly stored on fibres located at the centre of the muscle fascicules (Figure 3A, B and C). Despite the changes in myofibre CSA, there were no differences in CSA of type I fibres (Figure 3D and 3E; C: 232.7 ± 31.3; HF: 317.6 ± 38.9 CSA μm^2^ (P = 0.165)).

### Gene expression, biochemical and metabolic responses

**Figure 4:**
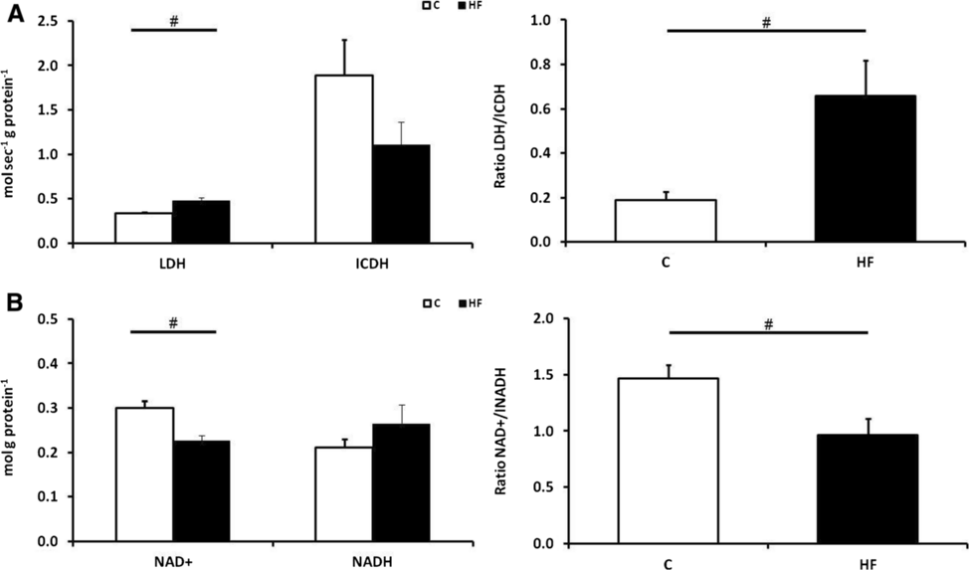
**Influence of maternal fat supplementation on skeletal muscle enzymatic activity.** **(A)** Enzymatic activities of total lactate dehydrogenase (LDH) and isocitrate dehydrogenase (ICDH) and their ratios in the *bicep femoris*. **(B)** Muscular concentrations and ratio of nicotinamide adenine dinucleotide (NADH) as well as its reduced form NAD^+^ on 7-day old offspring exposed to a control (white; n = 7) or high fat (black; n = 7) maternal diet throughout gestation. Bar graphs illustrate means ± SEM (#p < 0.05).

The gene expression and activities of enzymes involved in muscle metabolism were examined; the activity ratio of LDH (a pro-glycolytic enzyme) and ICDH (an indicator of global mitochondrial oxidation) were higher in the piglets born to mothers fed a HF diet compared to controls (Figure 4A). The ratio of NAD+/NADH, a marker of enhanced cellular oxidative capacity, was significantly lower in the HF group (Figure 4B).

**Figure 5:**
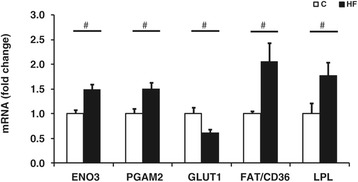
**mRNA abundance of Enolase 3 (ENO3), Phosphoglycerate mutase 2 (PGAM2), Glucose transporter 1 (GLUT1), Fatty acid translocase (FAT/CD36) and Lipoprotein lipase (LPL) in the** ***bicep femoris*** **of 7-day-old offspring exposed to a control (white; n = 7) or high fat (black; n = 7) maternal diet throughout gestation as determined by real time PCR.** Bar graphs illustrate means ± SEM (#p < 0.05).

The *biceps femoris* muscle of offspring born to HF fed mothers demonstrated raised expression of the genes ENO3, PGAM2, FAT/CD36 and a reduction in GLUT-1 (Figure 5), but there were no differences in either GLUT-4 (C = 1.0 ± 0.1; HF = 1.5 ± 0.5 (P = 0.12)) or CPT-1 (C = 1.0 ± 0.1; HF = 1.2 ± 0.1 (0.66)). Consistent with the increases in pro-glycolytic activity, the muscle samples obtained from HF offspring exhibited a higher concentration of glycogen compared to controls (C = 20.3 ± 2.4; HF = 61.8 ± 16.0 mg g tissue^−1^ (P = 0.007)). However, there were no differences between the groups for intramuscular triglyceride (C = 16.7 ± 1.6; HF = 23.8 ± 3.3 mg g tissue^−1^ (P = 0.31) or protein content (C = 39 ± 3; HF = 41 ± 2 mg g tissue^−1^ (P = 0.624)).

### Fatty acid content and lipid metabolism

The proportion of n-6 to n-3 phospholipids and linoleic acid (C18:2n6C) to α-linoleic acid (C18:3n3) (p < 0.01), were all higher in the HF offspring than controls (Table 2) as was the percentage of arachidonic acid (C20:4n6) and ratio of C20:4n6 to C20:3n6, a surrogate of 5Δ denaturase activity [34]. In contrast, the amount of C18:3n3 was reduced in offspring born to HF sows (Table 2). The proportions of a majority of non-essential (saturated and monounsaturated) fatty acids were similar between groups with the exception of arachidic acid (C20:0) in the HF group, which was higher than controls (Table 2).

**Table 2:** **Fatty acid composition of muscular phospholipids as measured in the piglets at 7 days of age after maternal high fat (HF, n = 7) or control (C, n = 7) feeding**

	**Group**	**C**	**HF**	**p**
***Non-essential fatty acids***				
**saturated**				
Palmitic Acid (C16:0)		21.5 ± 0.7	22.9 ± 0.8	
Stearic Acid (C18:0)		6.50 ± 0.27	6.92 ± 0.15	
Arachidic Acid (C20:0)		0.08 ± 0.02	0.23 ± 0.05	<0.02
C18:0/C16:0 (elongase activity indices)		0.30 ± 0.01	0.30 ± 0.01	
***Essential fatty acids*** **n-6**				
Linoleic Acid (C18:2n6c)		15.4 ± 0.9	14.9 ± 0.3	
Eicosatrienoic Acid (C20:3n6)		0.32 ± 0.01	0.33 ± 0.02	
Arachidonic Acid (C20:4n6)		2.14 ± 0.17	2.89 ± 0.24	<0.03
SUM n-6		17.9 ± 0.93	18.2 ± 0.47	
C20:4 n6/C20:3 n6 (Δ5 activity indices)		6.68 ± 0.58	8.84 ± 0.58	<0.01
***Essential fatty acids*** **n-3**				
α-Linolenic Acid (C18:3n3)		1.09 ± 0.12	0.79 ± 0.04	<0.02
Eicosatrienoic Acid (C20:3n3)		0.14 ± 0.03	0.08 ± 0.03	
Eicosapentaenoic Acid (C20:5n3)		0.11 ± 0.04	0.11 ± 0.04	
Docosahexaenoic Acid (C22:6n3)		0.34 ± 0.03	0.42 ± 0.04	
SUM n-3		1.67 ± 0.12	1.40 ± 0.07	
C18:2 6nC/C18:3n3		14.5 ± 1	19.3 ± 1.1	<0.01
Ratio n-6/n-3		10.7 ± 0.34	13.1 ± 0.53	<0.01

Values are means ± SEM. C: control; HF: high fat.

## Discussion

The objective of this study was to explore the potential consequences of fat supplementation of the maternal diet during gestation on skeletal muscle composition in the neonate – a period of intense energy use in the pig. We have demonstrated that the transition from oxidative to glycolytic muscular metabolism was enhanced in offspring born to fat supplemented sows. This was accompanied with changes in muscle phospholipid composition; namely an increase in arachidonic acid and a decrease in α linolenic acid leading to an increase in the n6/n3 ratio and an increased neonatal growth rate in the absence of any effect on offspring birth weight.

We hypothesise that muscle metabolism was reset in utero as a consequence of an increased supply of energy from the mother ultimately leading to improved glycolytic and lipogenic capacity, thereby promoting myofibre development during early lactation. Although neither individual skeletal muscles nor total muscle mass was assessed due to the limited space and time available during pig sampling within a normal commercial pig unit, we have previously shown that total muscle mass is not influenced by body weight after one week of age [35]. The progeny of fat supplemented sows may have experienced an improvement in the utilization of linoleic acid for conversion to arachidonic acid potentially activating prostaglandin production, an essential pathway for muscular development, although we did not observe differences in expression of cyclo-oxygenase (COX) 1 or 2 in the muscle from offspring [2,36,37]. To date, much of the developmental programming research involving increased fat consumption has investigated the effects of maternal obesity [8,38] but has not examined the effects of macronutrient replacement in an isocaloric manner in a large mammal, such as the pig. Only limited amounts of fatty acids cross the porcine placenta, so their contribution to fetal and muscular development is normally minimal [39]. Our dietary manipulation may have promoted a more efficient mobilization of maternal body fat, reflected by the raised maternal HDL which would allow a constant supply of glucose to the growing fetuses [40], for which glucose is a primary substrate for myogenesis [41]. Piglets born to mothers exposed to a fat supplemented diet grew faster up to 7 days and exhibited a proportional expansion of myofibres and a metabolic maturation toward glycolysis, in the *biceps femoris* similar to the findings of Jean and Chiang [42]. Histological analysis, however, failed to indicate which muscle fibre types were affected but decreased lipid staining was found within fibres located in the periphery of the fascicule, in parallel to a substantial increase in muscular glycogen, suggesting more efficient muscle metabolism [43]. Although we cannot exclude reduced carbohydrate having a role in regulating the changes observed, as summarised in a systematic review of animal studies [44] which have adopted fat feeding to mothers, our findings are supportive of the concept that fat is the main nutrient responsible.

Enhanced whole-body growth requires extra nutrition in order to meet the increased metabolic demands, especially within skeletal muscle [45]. An increase in nutrient flux would reset the cellular ratio of NAD^+^ to NADH thereby potentially increasing the concentration of NADH, which in turn, would inhibit activity of ICDH to decrease the influx of glycolytic metabolites through the mitochondria, these findings were observed in the fat supplemented offspring [41,46]. Additionally, the increase in mRNA expression of genes for proteins involved in glycolysis, such as ENO3 and PGAM2, together with greater GLUT-1 (which is independent of insulin) and LDH enzymatic activity are all indicative of greater glucose consumption [47]. Other indicators of muscular development, such as the muscular protein content, total triglyceride content and the mRNA expression of GLUT-4 were unaffected, emphasising that other components of muscle metabolism were unaffected.

### Can maternal diet modify the muscular incorporation of essential fatty acids in the neonate?

We also observed differential effects of maternal fat supplementation upon muscle fatty acid composition of the offspring, in particular n-6 and n-3 fatty acids. These essential fatty acids are crucial components of the cellular membrane, affecting structural and regulatory properties that need to adapt to changes in fatty acid supply [48,49]. There is indirect evidence of a different pattern of muscular incorporation of two essential fatty acids, arachidonic acid (C20:4n6) and α-linoleic acid (C18:3n3). Both of these fatty acids are involved in several metabolic pathways associated with muscular development and insulin signalling [8]. α-linolenic acid itself can repress the actions of arachidonic acid, it appears likely that reduced α-linolenic acid is responsible for the increased proportion of arachidonic, and may facilitate further muscle growth [50]. The fatty acid composition of skeletal muscle is also influenced by lipid binding proteins such as LPL and CD36/FAT, which allow circulating triglycerides to be hydrolyzed and free fatty acids to be accumulated [48,51]. Expression of both these genes was increased in fat supplemented offspring, suggesting these processes were enhanced.

## Conclusions

Isocaloric replacement of starch with palm oil in the diet of pregnant sows has no effect on birth weight but does promote the ability of offspring to differentiate and develop muscle fibres of the *biceps femoris* by increasing glycolytic capacity [52]. This pattern of growth is sustained by an increase in cellular energy uptake and usage as well as differential activation of elongases and desaturases, that could affect myofibre metabolism [49]. These adaptations, which remain to be quantified in the long-term, may be useful to improve piglet survival.

## Methods

### Materials and methods

All animal procedures described in this manuscript were approved by the Ethics Committee for Animal Experiments of the Animal Sciences Group of Wageningen Research Centre, and conducted at Schothorst Feed Research in the Netherlands. All laboratory procedures were carried out at The University of Nottingham under the United Kingdom code of laboratory practice (COSHH: SI NO 1657, 1988).

### Animals and diets

Fourteen Yorkshire X Landrace sows were artificially inseminated, then randomly allocated to one of the two gestational diets, either a commercial control diet (C; n = 7) or a high fat diet generated by supplementing the feed with palm oil (“high fat” HF; n = 7). All sows were between second and sixth parity and equally distributed between dietary groups with a mean parity of 3.8 ± 0.24 and fed to meet their net energy (NE) requirements during pregnancy (*i.e.* from 0 to 70 days of gestation 25.1 MJ NE/day and from 70 to 110 days of gestation 32.6 MJ NE/day). The caloric distribution of macronutrients for each diet was: control 64% starch, 11% fat and 25% protein; high fat 33.8% starch, 40.7% fat and 25.5% protein (Table 3). The feed provided to the HF group contained 50% more linoleic acid (C18:2n6) and 90% more saturated fat in the form of C16:0 and C18:0. In addition, the diets were supplemented to meet adequate essential amino acid, fatty acid, vitamin and mineral needs.

**Table 3:** **Composition and quantity of the experimental diets with alterations in macronutrient ratio**

**a) Maternal dietary composition (%)**				
		**Gestation diet**		**Lactation**
**Percentage**		**Control**	**Fat substituted**	
**Tapioca**		28.1	3.1	-
**Rapeseed meal**		10.0	10.0	4.0
**Sunflowerseed meal**		4.0	4.0	2.0
**Soybean hulls**		13.0	17.0	2.0
**Sugar beet pulp**		10.0	10.0	-
**Palm oil**		-	6.6	3.1
**Soybean oil**		0.5	0.5	0.97
**Maize**		-	-	10.0
**Sugar beet pulp**		-	-	2.0
**Soybean meal**		-	-	11.7
**Wheat**		10.0	10.0	26.4
**Barley**		10.0	10.0	15.0
**Wheat middlings**		15.0	15.0	15.0
**Molasses**		4.0	4.0	4.0
**Mono calcium phosphate**		0.22	0.22	0.48
**Salt**		-	-	0.37
**Limestone**		0.50	0.60	1.56
**Premix vit. and min.**		0.5	0.5	0.5
**Lysine (25**%**)**		0.17	0.17	-
**Lysine-HCl (L, 79**%**)**		-	-	0.17
**Threonine (L, 98**%**)**		-	-	0.02
**Phytase**		0.5	0.5	0.5
**Threonine (15**%**)**		0.01	0.01	-
**Sodium bicarbonate**		0.6	0.6	0.16
**Basic Feed allowance**		107.1	92.8	100.0
**b) Summary of major dietary components of the maternal diet and net energy. NB these nutrient levels were present in 1.07 Kg of the control diet and 0.92 Kg of the HF diet leading to a 15% reduction in intake in the HF diet. These diets were therefore isocaloric.**				
		**Gestation diet**		**Lactation**
	**Nutrients**	**Control**	**Fat substituted**	
**g/Kg**	**Ash**	64	52	61
**g/Kg**	**Crude protein**	123	122	161
**g/Kg**	**Crude fat**	25	89	63
**g/Kg**	**Crude fibre**	120	121	52
**MJ/Kg**	**Net energy**	9.3	9.3	9.5
**c) Feed quantities provided**				
		**Feed quantity during gestation (Kg/day)**		
		**Day 0-40**	**Day 40-70**	**Day 70-110**
**Control** (n = 8)		2.89	2.89	3.75
**Fat substituted** (n = 8)		2.51	2.51	3.26

The sows were weighed on 0, 40, 70 and 108 days of gestation prior to feeding. On 0, 40 and 108 days of gestation fasting blood samples were taken from each animal. The plasma was extracted in K^+^EDTA coated tubes, the samples were immediately separated by centrifugation (3000 g for 10 minutes at 4°C) and stored at −80°C until analysis.

From day 110 of gestation (mean caloric intake ≈ 27.3 MJ/day) and throughout lactation (59 MJ/day) all the sows were fed an identical diet sufficient to meet their energy requirements. Sows gave birth naturally in farrowing crates and all piglets were weighed after birth when litter size plus any still births were recorded. All piglets remained with their mothers and were allowed to suckle *ad libitum*. In all sows, milk was sampled, after milk let down via an intravenous oxytocine injection (10 I.U./mL; Eurovet Animal Health, Bladel, The Netherlands) into the ear on day 2 of lactation. At 7 days after parturition, the median birth weight offspring (C: 6 females, 1 male; HF: 4 females, 3 males) in each litter were weighed, a blood sample collected, the piglet sedated with 10% ketamine and then euthanized with (50 mg/kg) T-61 (Intervet, Boxmeer, Holland) followed by exsanguination.

### Laboratory procedures

#### Plasma and milk analysis

Plasma high-density lipoprotein (HDL), low-density lipoprotein (LDL) and glucose were determined by colorimetric assays and measured using an Imola RX automated apparatus (Randox laboratories Ltd. Co, Antrim, UK). The fat, protein and lactose concentrations in fresh milk were determined by infared analysis, using a Fourier Transform InfaRed (FTIR) interferometer (MilkoScan™, Foss Electric, Hillerød, Denmark).

### Muscle sampling

The *biceps femoris* is a morphologically and functionally distinct skeletal muscle which forms part of the hamstring muscle group and is located on the posterior thigh. Samples were removed from the centre of each muscle to standardize sampling location and immediately frozen in liquid nitrogen and subsequently stored at −80°C until analysis was performed. All homogenisation carried out during this study was performed using a gentleMACS™ closed homogeniser (Miltenyi Biotec Ltd., Surrey, UK).

### Enzymatic kinetics analysis

The lactate dehydrogenase (LDH) and isocitrate dehydrogenase (ICDH) total enzymatic assays were adapted from protocols obtained from SIGMA (http://www.sigmaaldrich.com/life-science/metabolomics/enzyme-explorer/learning-center/assay-library.html). Briefly, the muscular extract used (0.1 g) was homogenised in 4 ml buffer containing 0.25 M sucrose, 0.2 mM EDTA and 0.1 mM Tris (pH7.5). All samples were centrifuged at 6000 g for 15 minutes at 4°C and the supernatant diluted 1:2 with homogenisation buffer for use in subsequent assays. Total LDH enzymatic activity (EC 1.1.1.27) was determined in 1 μl of the muscle homogenate to which 10 μl NADH (0.33 mM) and 290 μl of reaction buffer (2 mM sodium pyruvate, 50 mM TEA, 5 mM EDTA) (Sigma-Aldrich Co LLC, Gillingham, UK) were added. Similarly, total ICDH enzymatic activity (EC 1.1.1.42) was determined in 7.5 μl of the muscle homogenate to which 270 μl of ICDH reaction buffer (38.9 mM Na2HPO_4_, 0.5 mM MnCl_2_ (4H_2_O), 0.05% βNADP) and 1 μl of (0.4 M) isocitrate (Sigma-Aldrich Co LLC, Gillingham, UK) were added. Enzymatic activities were measured by changes in absorbance at 340 nm at 28°C on a 96-well spectrophotometer (BIO-TEK Instruments Inc., Vermont, USA). The total LDH and ICDH activity were expressed as oxidised moles of their respective subtracts per second per gram of protein. The kinetic data were analysed by nonlinear regression using a method based on the principles of the Michaelis-Menten equations [53].

### Muscle composition

Total protein of the muscle homogenates was determined using a commercial kit (Bio-Rad Laboratories Inc. Hemel Hempstead, UK) based on the Bradford method [54] on. The results were corrected to the dissected muscle weight obtained from each offspring.

Glycogen content was determined using a method developed by Dalrymple and Hamm [55]. The concentration of glucose released from this reaction was determined by a colorimetric commercial assay (Randox Ltd., County Antrim, UK) and the results were expressed as a concentration of milligrams of glycogen per gram of tissue dissected.

NAD+/NADH ratio was determined using a colorimetric commercial kit (Sigma-Aldrich Co LLC, Gillingham, UK) and normalised to the quantity of tissue dissected i.e. moles per gram of protein.

Triglyceride content was assessed using the Folch method [56] followed by colorimetric commercial assay (Randox Ltd., County Antrim, UK) and results expressed in milligrams per gram of tissue dissected.

Phospholipid composition was determined by gas-chromatography in which the chloroform phase was evaporated by applying a nitrogen stream and then 2 ml of hexane was added to each sample. The phospholipids were transmethylated by adding 40 μl of methyl acetate and 40 μl of methylation reagent (30% sodium methoxide, 4.1 ml methanol; Fisher Scientific Ltd. Loughborough, UK) to each reaction and left to react for 10 minutes at room temperature. This was followed by adding 60 μl of termination reagent (0.2 g dried oxalic acid and 6 ml diethyl ether) and methyl ester residues were extracted by adding 200 mg of calcium and followed by short centrifugation. The fatty acid methyl esters were then injected (split ratio 50:1) into a gas chromatograph (GC 6890; Agilent technologies Ltd, Stockport, UK). Separation of fatty acid methyl esters was performed with a Varian CP-88 (Crawford Scientific™ Ltd., Strathaven, UK) capillary column with hydrogen as carrier gas. Oven temperature was programmed from 59°C to 100°C at 8°C per min, then to 170°C at 6°C per minute and held for 10 minutes. The temperature of the injector and detector were set at 255°C and 250°C. The fatty acid methyl esters were identified by comparing the retention times with a fatty acid methyl esters standard (Sigma-Aldrich Co LLC, Gillingham, UK) and the area percentage in moles were used for the statistical analysis.

### Histological analysis

Immediately after removing the muscle samples from −80°C storage, approximately 1 cm^3^ of frozen tissue was sectioned and left to equilibrate overnight at −20°C. The next day, each sample was embedded on a cryostat metal plate in such a manner as to set the muscle fibres perpendicular to the cutting blade. The mounted samples were cut into 10 μm thickness in a pre-equilibrated at −20°C Leica Cryostat (Leica Microsystems Ltd., Milton Keynes, UK). The slices were then mounted on polysine histological slides (Menzel-Glaser, GmbH & Co. Braunschweig, Germany) and allowed to equilibrate to room temperature prior to staining.

Haematoxylin and eosin staining was determined on sections placed in filtered 0.1% Harris’ haematoxylin for 3 minutes and gently rinsed in cool running water to extract excessive stain. This was followed by further rinsing the sections in acidic alcohol and cool running water before transfer to eosin for 2 minutes. After staining, the slides were dipped in distilled water, dehydrated back through a graded series of alcohol concentrations and placed in xylene for 5 minutes. The stained sections were mounted with a coverslip in resin base medium and finally incubated overnight at room temperature.

Immunostaining of slow myosin (MyHC) was determined using anti-slow myosin pig specific MyHC antibody (Sigma-Aldrich Co. LLC., Gillingham, UK) at 1:4000 dilution. The staining was carried out on the Bond Max histology system using the Bond Polymer Refine Detection System (Vision Biosystems, Mount Waverley, Australia) and Bond software version 3.4A. Briefly, slides were stained as follows: 15 minutes with primary antibody, 8 minutes with secondary antibodies, 10 minutes with 3,3 diaminobenzine and counterstained with hematoxylin. A negative control slide in which the primary antibody stage was excluded was included with each batch.

Oil red O histochemical lipid staining was used to assess the intramyofibre lipid content which detects cellular fat droplets as a light red tint. Briefly, the 10 μm cryosections were placed in a Coplin jar containing 60% isopropanol for 15 minutes. Immediately afterwards, the sections were incubated for 20 minutes in a freshly-prepared solution containing 0.5% w/v oil red-O/isopropanol (Fisher Scientific Ltd. Loughborough, UK) diluted in a 1% w/v solution of dextrin. Thereafter, the stained samples were immersed for 1 minute in 60% isopropanol and then rinsed in cool running water. This was followed by counterstaining of the muscular fibres with Harris’ haematoxylin for 3 minutes, followed by a quick rinse in cool running water. In addition, the slides were dipped 3 times in Scott’s tap water and fast dried. Finally, a drop of 50% v/v glycerol-water solution was placed over the samples in order to preserve the lipids and the coverslip was sealed by applying an acetone-based nail polish.

Images of the sections were captured using a high performance CCD camera connected to a Leica universal microscope at 20 x magnification (Leica Microsystems Ltd., Milton Keynes, UK). From each image, 10 random pictures were taken for analysis. The mean cross-sectional fibre areas obtained from each image were analysed by dividing them into 12 equal fields and choosing one by random in which at least 150 fibres were measured through the use of image analysis software (Image-pro plus; MediaCybernetics, Bethesda, USA). Irregular-shaped fibres were excluded using a custom-made macro that drew a ring over each myofibre; filtering criteria were applied to reject regions with a CSA < 50 μm^2^ or > 5600 μm^2^ or regions with circularity (approximation diameter of an ellipse) of <0.3 or >1.0. For type I fibre analysis, a function was incorporated in our custom-made macro to detect the colour intensity of the positive-stained fibres. Finally, for the Oil red O analysis, the colour density threshold was adjusted to distinguish all the fat droplets on a single fibre. Only the droplets with an area > 0.4 μm^2^ and < 1.5 μm^2^ in those encircled fibres were included in this analysis.

### Gene expression

Total RNA was extracted from 100 mg skeletal muscle using a commercially available kit (Qiagen Ltd., Crawley, UK), which included a DNA purification step. The amount and purity of the extracted RNA was determined using a Nanodrop (Thermo Fisher Scientific Ltd. Leicester, UK). 1 μg of total RNA was reverse transcribed using a Thermocycler (Thermo Fisher Scientific Ltd. Leicester, UK). Quantitative PCR (qPCR) was performed using a Roche lightcycler 480 thermocycler and SYBR technology (Roche, Burgess Hill, UK). For quantification of gene expression we applied the comparative C^t^ method, which was normalised by applying a geometric mean of 3 endogenous housekeeping genes (18 s rRNA, β actin and cyclophillin) [57]. The genomic data is expressed in this study as a ratio to the commercial control animals.

The qPCR primers were designed based on known porcine sequences published on the Genbank using public online software (Primer3) on intra-exonic boundary sequences where possible. A standard curve was included and the samples were run in duplicate as well as having the appropriate positive and negative controls. The primers used were purchased from Eurofins MWG Operon GmbH. (Ebersberg, Germany) and validated as described in previous publications [58]. The following porcine-specific oligonucleotide forward (F) and reverse (R) primers were used:

ENO3: F:GAGCTGGATGGGACAGAAAA–R:GCAATGTGACGGTAGAGTGG; PGAM2:F:GATCAAGGCAGGCAAGAGAG–R:ACATCCCTTCCAGATGCTTG; FAT/CD36: F:TGAAAGAAGCAGGTGCTGAA–R: AGGACTGCTCCCAATGACAGC; Primers for cyclophilin, 18S, β-actin, LPL, GLUT-4 and CPT1 have previously been published [58-60].

### Statistical analysis

Statistical analysis of the data was performed using SPSS® statistics software (v 16.0 for Windows; IBM, Chicago, USA). The data was tested for normality by applying the Kolmogorov test and controlled by observation of its Gaussian distribution. Depending on the results of the previous tests, the data was analysed by applying Student’s unpaired *t*-test and linear regression analysis with Benjamini & Hochberg False Discovery Rate correction; otherwise the non-parametric Mann Whitney test and Spearman Rank correlation were used. In all cases, the results are given as mean ± SEM, p < 0.05 was considered as statistical significant.
